# Pediatric Attention-Deficit/Hyperactivity Disorder in Louisiana: Trends, Challenges, and Opportunities for Enhanced Quality of Care

**DOI:** 10.31486/toj.18.0103

**Published:** 2019

**Authors:** Rohail Kumar, Mary Margaret Gleason

**Affiliations:** ^1^ Tulane University School of Medicine, New Orleans, LA; ^2^ Department of Psychiatry and Behavioral Sciences, Tulane University School of Medicine, New Orleans, LA; ^3^ Department of Pediatrics, Tulane University School of Medicine, New Orleans, LA

**Keywords:** *Attention deficit disorder with hyperactivity*, *Louisiana*, *pediatrics*, *primary health care*

## Abstract

**Background:** Attention-deficit/hyperactivity disorder (ADHD) is a common pediatric condition with significant developmental, social, educational, and safety implications. The American Academy of Pediatrics has developed guidelines to support quality care of children with ADHD, but studies demonstrate that the guidelines are variably followed.

**Methods:** This review highlights patterns of diagnosis and treatment of children with ADHD nationally and in Louisiana and provides examples of system- and practice-level opportunities to improve adherence to quality standards.

**Results:** Possible contributors to the higher prevalence of ADHD and medication use in Louisiana compared to the nation are specialty workforce shortages, factors in the educational system, and factors associated with race and geography. Innovative system approaches have been developed to address workforce shortages and training limitations. Practice-level innovations include improving the use of validated measures, offering adequate scheduling, and identifying relevant resources and sharing the information with families.

**Conclusion:** Despite the availability of evidence-based recommendations and resources, significant opportunities exist to provide enhanced ADHD care at the primary care level, especially in Louisiana where the high prevalence of some risk factors for ADHD and the high rates of ADHD and medication prescriptions have been noted nationally and at the state level. Attention to these factors can potentially help address these disproportionalities. Additionally, innovative models of training and collaboration in pediatrics are imperative. Pediatric clinicians, mental health providers, and families can work together to increase awareness about the needs of children and families affected by ADHD in medical, educational, and policy arenas and move the system forward for children.

## INTRODUCTION

**Figure 1.**
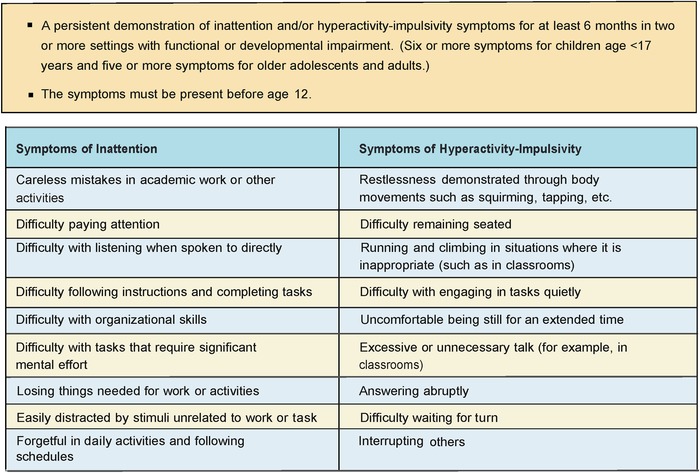
**Overview of *Diagnostic and Statistical Manual of Mental Disorders, Fifth Edition* diagnostic criteria for attention-deficit/hyperactivity disorder.^1^**

Attention-deficit/hyperactivity disorder (ADHD) is one of the most common psychiatric conditions in children and adolescents and contributes to significant academic and functional impairment. Diagnosis is primarily clinical, involving confirmation of symptoms and differentiation from other causes of hyperactivity and inattention based on the criteria in the *Diagnostic and Statistical Manual of Mental Disorders, Fifth Edition* (DSM-5) (Figure 1).^1^ ADHD can present with predominantly inattentive symptoms, with hyperactive/impulsive patterns, or with both; each subtype requires the presence of at least 6 of the 9 criteria. The symptoms must be present for 6 months in at least 2 different settings and must cause impairment in social, academic, or occupational functioning. Untreated, ADHD can be associated with significant problems in academics, social domains, and health, but treatment can be effective and reduce impairment in children and youth.

Parent-reported surveys from 2011^2^ and 2016^3^ suggest that 9.4% to 11% of children in the United States had received an ADHD diagnosis, with a 42% increase in the rates of diagnosis between 2004 and 2011.^2^ Additionally, rates reported in the United States are much higher than those reported in other developed countries^4^ and higher than the worldwide prevalence.^5^ Results from a large epidemiologic study from the United States suggest a prevalence (8.6% to 10%) similar to those reported by parent surveys.^6^ However, the study identified that a substantial proportion of children taking ADHD medications did not meet the criteria for ADHD, and only about half of children who met the criteria for ADHD had been previously identified.^6^ These differences highlight the importance of attending to patterns of identification, diagnosis, and treatment. Discussion of ADHD in Louisiana is important because Louisiana leads the nation in rates of pediatric ADHD medication use.^2,7^

This review describes trends in ADHD diagnosis and management nationally and in Louisiana and describes opportunities for adherence to best-practice guidelines.

## TRENDS IN ATTENTION-DEFICIT/HYPERACTIVITY DISORDER DIAGNOSIS AND MANAGEMENT

### American Academy of Pediatrics Attention-Deficit/Hyperactivity Disorder Guidelines

**Figure 2.**
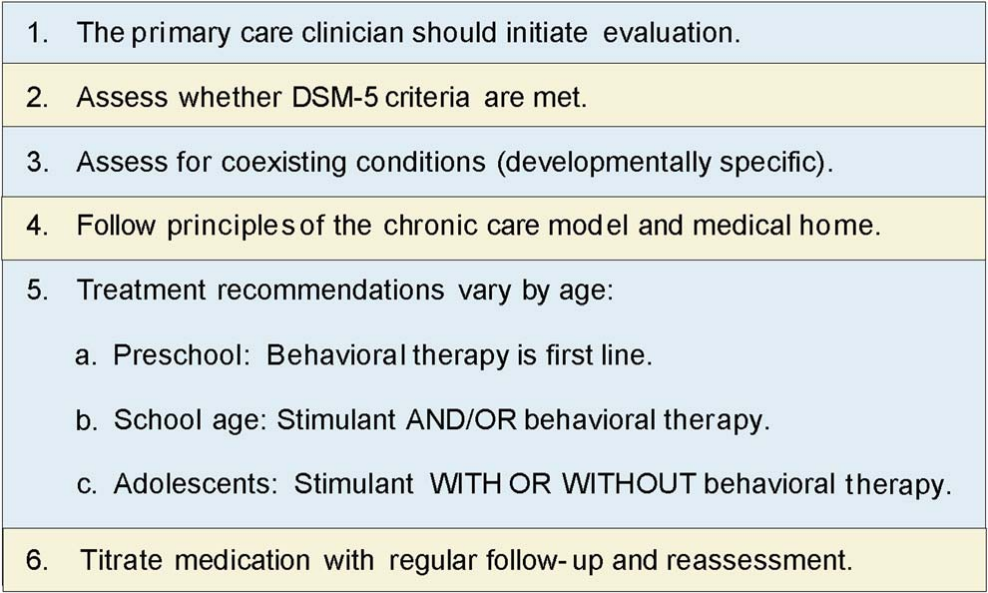
**Key action statements from the American Academy of Pediatrics attention-deficit/hyperactivity disorder guidelines.^8^** DSM-5, *Diagnostic and Statistical Manual of Mental Disorders, Fifth Edition*.

In 2011, the American Academy of Pediatrics (AAP) published an update to the best-practice guidelines for the evaluation and management of ADHD in children and adolescents with a focus on developmentally specific recommendations (Figure 2).^8^ The guidelines include age-specific treatment recommendations for preschool-age children (4 to 5 years), elementary school–age children (6 to 11 years), and adolescents (12 to 18 years).^8^ Familiarity with these guidelines is important for primary care clinicians caring for children who have any behavioral concerns.

The AAP and the Louisiana Task Force on ADHD,^7^ established in 2014, have emphasized the importance of using standardized tools such as ADHD rating scales as part of the diagnostic process and of tracking the response to treatment.^8^ Validated rating scales are particularly valuable for eliciting information from teachers to demonstrate the presence of symptoms and impairment in more than one setting.^9^ Rating scales allow for comparisons across children, across reporters, and across time and assist in laying a foundation for managing ADHD using a chronic care and medical home model as recommended by the AAP ADHD guidelines.^8^

For preschool-age children diagnosed with ADHD, the AAP recommends behavioral therapy as the first-line treatment.^8^ In this age group, the AAP and other practice guidelines recommend consideration of medication if symptoms persist after an adequate trial of behavioral therapy or if behavioral therapy is not available.^8,10^ For children 6 to 11 years, the AAP recommends prescribing stimulant and/or behavioral therapy, and for adolescents 12 to 18 years, the recommendation is stimulant with or without concurrent behavioral therapy.^8^

In young children, behavioral therapy focuses on parent management training strategies. In older children, behavioral therapy includes a combination of family-focused strategies to support a child's organization and impulse control and the development of self-regulation skills and organizational skills in the child.

### National Trends

Despite the 2011 AAP guidelines, significant variability in ADHD care persists. In a 2014 study of pediatricians across 50 practices, approximately 70% reported applying DSM-IV criteria when diagnosing children with ADHD, and approximately half used an adult-report measure (parent or teacher rating scale)^11^ as recommended by the guidelines. However, duration from starting a stimulant to first follow-up averaged 72 days,^11^ substantially longer than the recommended 30-day follow-up.^12^ Delayed follow-up can result in undertreatment with ineffective doses and prolonged exposure to side effects.

Despite recommendations to use validated measures, the Epstein et al 2014 study showed that only 10.8% of pediatricians used a parent follow-up ADHD rating scale to monitor treatment outcomes, and only 7.5% obtained teacher rating scales to monitor treatment outcomes.^11^ Further, while 93% of the children were receiving medications, only 13% received behavioral therapy. These findings suggest that patients in the 6- to 11-year-old age group—when stimulant and/or behavioral therapy is recommended—overwhelmingly received stimulants instead of behavioral therapy.

Information about ADHD treatment in preschool patients is limited, but after the publication of the AAP guidelines in 2011, rates of diagnosis of ADHD in preschoolers, which had been increasing, slowed.^13^ However, the proportion of preschoolers with ADHD who were treated with medication did not change.

### Louisiana-Specific Trends

**Figure 3.**
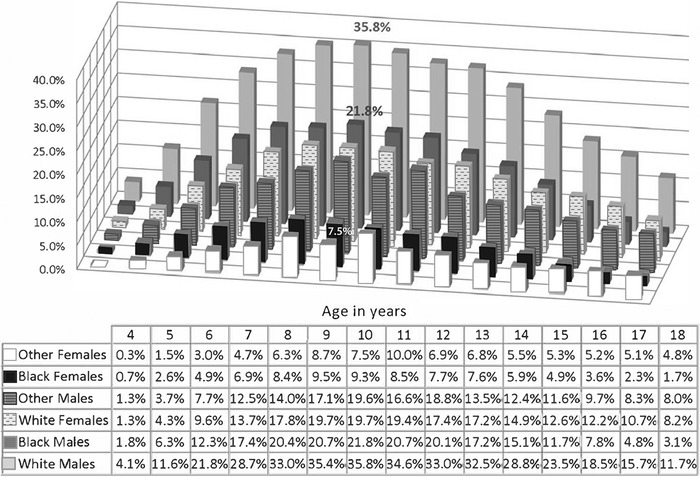
**Attention-deficit/hyperactivity disorder prescription rates in Louisiana (2013) by age, sex, and ethnicity.^7^**

In 2011, the Centers for Disease Control and Prevention (CDC)^14^ used data from the National Survey of Children's Health^2^ to show that 15.8% of children in Louisiana had been diagnosed with ADHD (compared to the national average of 11%), and 10.4% of children in Louisiana were taking medications for ADHD.^14^ Louisiana children covered by Medicaid receive more prescriptions for ADHD medications (12.9%) than the state average (10.4%), costing Louisiana Medicaid approximately $30 million.^7^ Furthermore, the rate of ADHD diagnosis for Louisiana boys is substantially higher than the national rate (19% vs 12%), with an even higher rate among boys covered by Medicaid (22%).^7^ Interestingly, the rate of ADHD diagnosis for girls in Louisiana does not differ substantially from the national rates. In Louisiana, white boys receive ADHD prescriptions at the highest rate of all youth which is comparable to national trends (Figure 3).^7^

Another identified factor in prescribing patterns is age compared to classroom peers, which has been demonstrated in Louisiana and internationally.^7,15,16^ Beginning at age 6 in Louisiana, children with birthdays in September (the youngest children in the class) are 26% more likely to have an ADHD prescription than children born in October,^7^ suggesting that classroom expectations influence treatment patterns.

**Figure 4.**
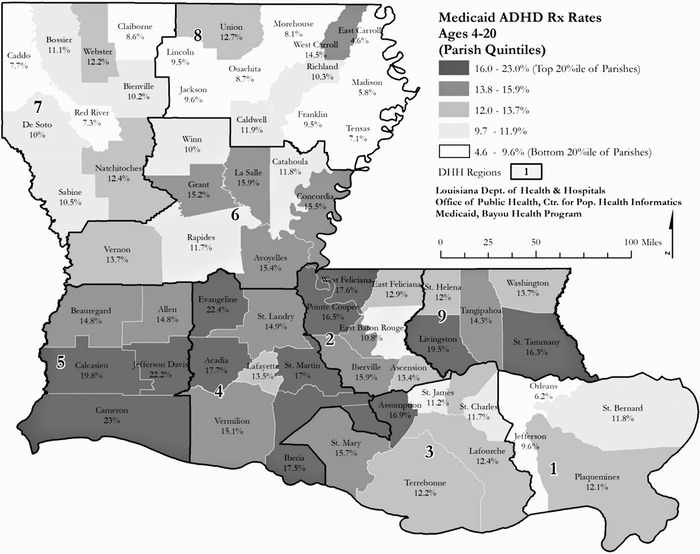
**Attention-deficit/hyperactivity disorder (ADHD) prescription rates in Louisiana (2013) by geographic region.^7^** DHH, Department of Health and Hospitals; Rx, prescription.

Geographic factors also seem to play a role in treatment decisions, with significant variability across the state, although the specific factors remain somewhat elusive. Generally, but with notable variability, the southern part of the state along the I-10/I-12 corridor (with the exception of New Orleans) showed higher rates of ADHD prescriptions than northern Louisiana (Figure 4).^7^ Hypothesized factors, including school districts, demographics in the region, insurance status (public vs private), and density of mental health providers, did not explain the variability, raising questions about the contribution to differences in trends from national data of other nonclinical factors such as variability in the diagnostic process.

Louisiana's patterns of diagnosis and prescribing raise an important question about why Louisiana has high rates of ADHD identification and, relatedly, high rates of ADHD prescriptions. Children in Louisiana may possibly be at higher risk for ADHD than children in other states. Consistent risk factors and correlates of ADHD in the literature can be categorized as genetic, prenatal, and environmental. The high inheritability of ADHD may result in increased rates of ADHD among populations that tend to stay in a geographic area. Prenatal exposures to substances including tobacco, alcohol, and illicit substances; slow fetal growth; and maternal stress are associated with developing ADHD^8^ and are seen in Louisiana at rates higher than the national rates, specifically, the incidence of low birth weight.^17^ Postnatal environmental risk factors for ADHD include adversity, stress, and exposure to toxins such as lead. Rates of exposure to adversities such as natural disasters, murder, and adverse childhood experiences occur at high rates in Louisiana.^18-20^ Louisiana's overall rate of exposure to elevated lead levels (1.3%) is similar to the national rate.^21^

**Figure 5.**
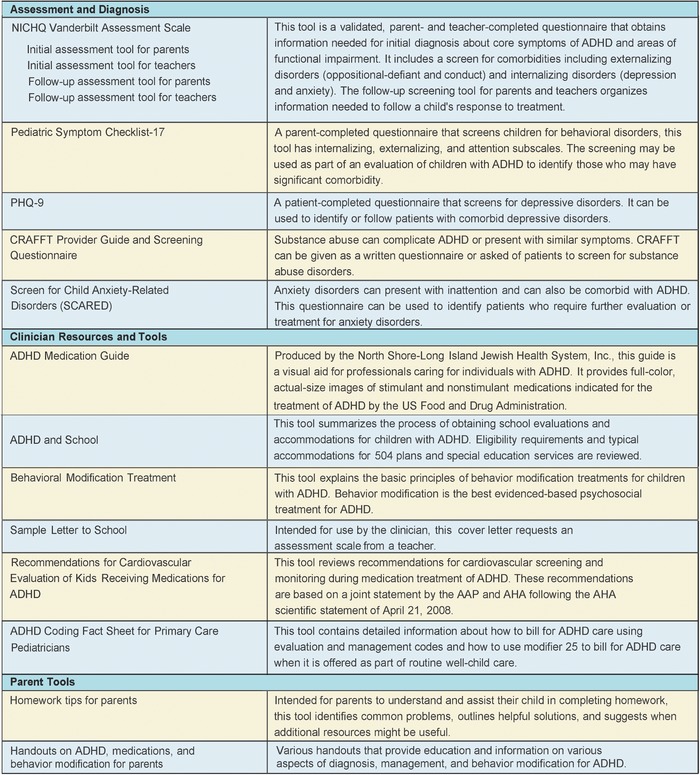
**Summary of the American Academy of Pediatrics (AAP) attention-deficit/hyperactivity disorder (ADHD) toolkit (second edition).** AHA, American Heart Association; CRAFFT, Car, Relax, Alone, Forget, Family/Friends, Trouble; NICHQ, National Initiative for Children's Health Quality; PHQ-9, Patient Health Questionnaire-9.

In 2014, the Louisiana Task Force on ADHD was established after the Louisiana legislature commissioned the Department of Health and Hospitals to address concerns about ADHD management. The task force developed recommendations focused on diagnostic accuracy, improved system coordination, and empirically supported prescribing practices, and the Department of Health and Hospitals sponsored a day-long teacher training focused on supporting children with ADHD.^7^ Additionally, the Louisiana Medicaid managed care organizations provided evidence-based treatment training for Louisiana clinicians and collaborated with the Louisiana Chapter of the AAP to purchase licenses for the AAP ADHD toolkit for all Louisiana providers.^22^ The toolkit provides evidence-based screening tools to help clinicians assess ADHD in children aged 4 to 18 years and specific screening tests to help clinicians identify comorbid conditions. The toolkit includes handouts and educational materials for both clinicians and parents that address the treatment of ADHD, monitoring, and follow-up and also includes tools designed to help families better understand ADHD (Figure 5).

## OPPORTUNITIES FOR ENHANCED ATTENTION-DEFICIT/HYPERACTIVITY DISORDER CARE

**Figure 6.**
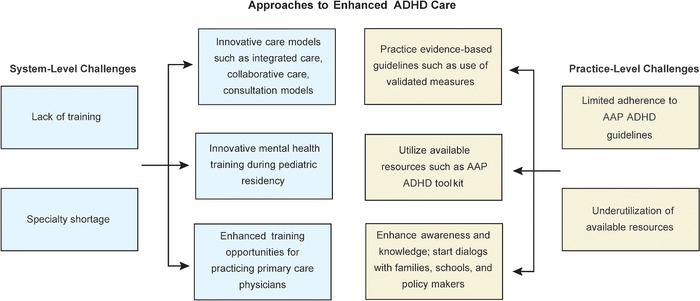
**Approaches to enhanced attention-deficit/hyperactivity disorder (ADHD) care.** AAP, American Academy of Pediatrics.

Trends and practice patterns nationally and in Louisiana suggest opportunities for enhancing the quality of ADHD care. At a system level, factors such as workforce availability, quality of training, and accessibility of innovative care models are important for enhanced care, while at the practice level, practicing evidence-based medicine and providing standardized care utilizing various resources and services are vital (Figure 6).

### System-Level Factors

#### Workforce Shortages and Training

Although noncomorbid mild to moderate ADHD can be managed in the primary care setting, children with complex presentations or more severe ADHD benefit from specialty evaluations and treatment to assess for comorbid conditions and/or to identify other causes for the clinical presentation.^8^ Shortages of specialty providers limit access to appropriate diagnosis and may result in inadequate or inappropriate treatments. The American Academy of Child and Adolescent Psychiatry (AACAP) estimates that ≥47 child and adolescent psychiatrists (CAPs) are needed for every 100,000 children.^23^ No counties in the United States achieve this goal, and most of the country is defined as a “severe shortage” area. Louisiana has approximately one-seventh of the required CAPs for the pediatric population at 7.17 CAPs per 100,000 children, and the CAPs are generally clustered in large population centers.^23^ Other specialties have workforce shortages as well. For example, according to the AAP membership directory, Louisiana has 5 developmental and behavioral pediatricians, principally in urban centers.^24^ Similarly, as of 2013, Louisiana ranked lowest for per capita clinical and school psychologists and also had a severe shortage of social workers.^25^ Overall, these shortages mean extensive wait times; limited access; and a burden on pediatric primary care, clinicians, and families.

Both the AAP and the AACAP recommend that primary care clinicians play the primary role in addressing common mental health issues such as ADHD and depression.^8,26^ However, pediatric residency training rarely prepares trainees sufficiently for this role. In a 2010 survey, fewer than half of graduating pediatric residents reported they had good to excellent competence in dealing with children's mental health.^27^ Further, 65% of practicing pediatricians reported in 2013 that they lacked training in children's mental health problems.^28^ One contributing factor may be that the faculty members who are training most pediatric residents have limited training or confidence in addressing mental health concerns themselves. To date, pediatric training does not require any clinical training exposure to child psychiatry, despite the expectation to learn to assess and to manage many psychiatric disorders.^29^ The required developmental and behavioral pediatrics rotation, which was mandated in 2000,^30^ may provide some training, but limited access to developmental and behavioral pediatricians means that the training remains variable.^31^

After residency training, continuing medical education opportunities are available to keep physicians up to date on trends and guidelines. These opportunities are generally designed as short didactic courses rather than interactive educational experiences.

#### Workforce and Training Innovations

Some efforts have been made to increase the workforce of specialists who can meet the needs of children with ADHD and related mental health concerns. The Triple Board Program^32^ and the Post Pediatric Portal Program^33^ are two innovative residency programs developed as alternative pathways to train physicians in child and adolescent psychiatry. The Triple Board Program, started in 1986, is a 5-year program that trains physicians who are eligible for board certification in pediatrics, psychiatry, and child and adolescent psychiatry. The Post Pediatric Portal Program is a 3-year program to train board-eligible pediatricians in general and child and adolescent psychiatry. In Louisiana, the Triple Board Program at Tulane University has trained 15 residents since 2010, more than half of whom practice pediatric psychiatry along the Gulf Coast, principally in the Greater New Orleans area. While these innovative training programs contribute valuable and well-trained specialists, the shortage remains substantial. Several states, including Louisiana, have offered loan repayment programs for healthcare providers trained in psychiatry, with the goal of addressing local workforce shortages.^34^

Efforts also address the training of pediatric residents. The AAP has developed training modules about several mental health topics, motivational interviewing, and the ADHD toolkit, most of which are publicly available on the AAP website (www.AAP.org). The availability of these training modules allows all residency programs, including those with limited access to specialists, to provide quality training on these topics.

Around the country, residency programs are developing creative approaches to increase interactions among pediatric residents and mental health specialists, including child and adolescent psychiatrists through required clinical rotations, elective rotations, and didactics.^35^ The pediatric residents at Tulane University have a 1-month required rotation in child and adolescent psychiatry to ensure their exposure to mental health programs in the community and to develop skills in interviewing, differential diagnosis, and primary care interventions for mental health concerns. The American Board of Pediatrics is also committed to improving mental health training in pediatrics and has convened several meetings with key stakeholders to develop innovative training opportunities.^36^

Innovative training efforts focused on pediatricians after residency include a program offered by the REsource for Advancing Children's Health (REACH) Institute in which primary care clinicians participate in an intensive course on child mental health, followed by ongoing longitudinal group case discussions.^37^ Project ECHO offers weekly mini-didactics and case discussions around the country and globally through telehealth connections; a growing number are focused on child mental health.^38^

#### Innovative Care Models–Collaboration, Integration, and Consultation

At least 19 states have statewide telehealth child psychiatry consultation programs.^39^ Unlike one-on-one direct services, telehealth consultation can influence the entire population cared for by a primary care clinician, not just the child being discussed, because the clinician can apply the new knowledge to similar clinical situations. Telehealth consultation offers a dynamic learning opportunity for primary care clinicians that potentially can help them manage patients with ADHD or other mental health concerns. Although the pediatric data are limited, long-term cost analyses in adults have shown significant cost savings: $1 spent on collaborative care saves $6.50 in direct healthcare costs.^40^ One study in adolescents with depression showed an overall net decrease in cost and improvement in the quality of life.^41^ Because consultation codes are not reimbursable in most states, most consultation programs are funded at the state level through Medicaid, state sources, federal grants, or philanthropic grants.

Data demonstrate that participation in the consultation is associated with increased use of mental health skills discussed in consultation^40^ and high rates of satisfaction. In Louisiana, collaborative models have limited scope but have been well received and show promise. The Tulane Early Childhood Collaborative (TECC) in the Greater New Orleans area and Project LAUNCH (Linking Actions for Unmet Needs in Children's Health), an Office of Public Health/Office of Behavioral Health project, offer interdisciplinary direct consultation at the pediatric office and telehealth consultation for young children by primary care clinicians.^42,43^ TECC has developed handouts and training about ADHD and is specifically focused on enhancing ADHD care through educational tools and quality improvement activities. Clini-cians at Louisiana State University provided collaborative treatment to patients in Louisiana parishes affected by the Deepwater Horizon oil spill and reported a significant decrease in psychiatric symptoms among the adults who received treatment.^44^ In addition, the primary care clinical staff reported an increase in their confidence and competence in evaluating and managing patients’ mental health needs in the future. While this study did not include treatment for children, a similar approach could be used to provide mental health care to children in areas that lack access to mental health care.

Other models involve the addition of lay care coordinators to pediatric practices to improve mental health outcomes in children. A 2010 study showed that the addition of case managers to pediatric teams facilitated communication between primary care clinicians and specialty services treating children with ADHD, resulting in improved clinical outcomes and parental satisfaction.^45^ Other studies have shown that using case managers who are trained to provide psychoeducation to parents and their children with ADHD leads to reduced family stress, improved provider diagnostic and management skills, and improved ADHD symptoms.^46,47^

To date, no data support that one approach may be better than the other. Future research can focus on whether the most successful models are those that are flexible, utilize local expertise, address community-specific needs, and work within existing healthcare systems.

### Practice-Level Opportunities

At the practice level, the use of validated measures, development of comprehensive assessment recommendations, scheduling changes, and adequate access to resources can influence the quality of ADHD care.

#### Validated Measures

Caregiver-report measures to diagnose ADHD in children are used in less than half of evaluations.^11^ Several barriers may influence the limited use of these measures in pediatrics. Pediatricians’ concerns about using checklists include time, resources, and the perception that checklists are too impersonal for use in practice.^48^ However, a 2017 study showed that using the Early Childhood Screening Assessment^49^ did not add to appointment time, and many providers felt that their practice was more efficient when using written measures.^50^

Pediatric clinicians can consider obtaining follow-up measures at regular intervals (for example, 1 to 2 months into the beginning of school and before the end of the school year) or in association with medication titration or symptom changes. The Healthcare Effectiveness Data and Information Set, which defines quality indicators for most insurers, sets the standard of 3 follow-up visits for children in the 10 months after initial diagnosis.^12^ This schedule may be a useful guide for when providers can use measures to follow up on symptoms and consider titration of medication doses.

Primary care clinicians may not want to rely solely on parents and children with ADHD to be the liaison between providers and the children's schools. To encourage the collection of information from teachers, one strategy is to include the clinician's office phone and/or fax number on the scale. Providing this information can lead to direct conversation if the teacher desires. School fax numbers may be available on websites, and keeping a list of these numbers for easy access can be a useful practice for primary care clini-cians. The AAP ADHD toolkit includes a letter for clinicians to send to teachers that explains why the teacher's input is valuable in guiding treatment plans; this information may increase some teachers’ motivation to complete and return the measure.

#### Assessment of Comorbid Conditions and Social Contributors to Attention-Deficit/Hyperactivity Disorder

Approximately two-thirds of children with ADHD have a comorbid condition.^51^ Primary care clinicians have limited time during pediatric visits, so procedures that support efficient data collection are useful. First, knowing a family history informs risk status. Children with a family history of ADHD, mood disorders, and substance use disorders should be considered at higher risk for developing ADHD or other disorders than children without such a family history. Identifying these disorders routinely during an initial visit with a family increases efficiency and may facilitate the early identification of disorders in children. Considering developmental status and learning problems is critical when a child presents with school or behavioral concerns. Conducting validated developmental screens routinely as recommended by the AAP can be helpful for the early identification of children at risk of learning problems. Additionally, every child presenting with emotional and behavioral concerns such as ADHD should be verbally screened for depression, anxiety, and PTSD. While the Vanderbilt Assessment Scale is well validated for identifying ADHD, its 37% sensitivity in identifying anxiety or mood problems is inadequate, and clinicians must use an additional measure or explicitly review symptoms of mood and anxiety as part of an ADHD assessment.^52^ For children older than 8 years, the Pediatric Symptom Checklist-17 can be used to explore a broad range of symptoms, including mood and anxiety.^53,54^ Other measures such as the Patient Health Questionnaire-9A^26,55,56^ or the Screen for Child Anxiety Related Emotional Disorders^57,58^ can be used in cases of suspected depression or anxiety, respectively. To explore environmental stressors, primary care clinicians can consider using tools such as the Safe Environment for Every Kid (SEEK),^59^ Well-Child Care Visit, Evaluation, Community Resources, Advocacy, Referral, Education (WE CARE),^60^ or Center for Youth Wellness Adverse Childhood Experiences Questionnaire.^61^ The Tulane Early Childhood Collaborative uses a version of the SEEK that is modified to include locally common traumatic events.^62^ Using such measures routinely elicits information efficiently and establishes a framework for biopsychosocial assessment that can be consolidated in future visits and further evaluations. The Screening Technical Assistance Resource (STAR) center is an initiative from AAP that provides healthcare professionals with evidence-informed technical assistance and resources to assist in implementing effective screening, referral, and follow-up recommendation.^63^ The website also includes an online tool finder to assist providers in finding and utilizing an evidence-based screening tool.^64^

#### Adequate Scheduling

The landmark multimodal treatment of ADHD study demonstrated that scheduling likely plays an important role in adequate treatment.^65^ In this study, a group of 579 children, aged 7 to 10 years, with ADHD combined type were assigned to 14 months of medication management (titration followed by monthly visits); intensive behavioral treatment (parent, school, and child components, with therapist involvement gradually reduced over time); the two combined; or standard community care (treatment by community providers). More frequent visits (8.8 vs 2.3 in a year) and longer appointments (30 minutes vs 18 minutes) were key differences between the medication management group and standard community care group, despite both groups receiving medication treatment.^65^ The standard community care group had the worst outcomes and received lower doses of stimulants, suggesting that the scheduling differences may have resulted in insufficient time to assess and effectively treat the symptoms.

Adequate visit schedules can be achieved by developing a patient registry for children with ADHD. A patient registry offers a way to track patients with special healthcare needs who should be followed at shorter intervals than the children who are healthy and developing typically. A patient registry, ideally embedded in an electronic medical record, can provide flags if a patient has missed or not scheduled appointments and may include reminders to contact the patient. Registries can also include ticklers to remind primary care clinicians or the team to send a symptom checklist to the child's school.

#### Resource Information

While many practices do not have a case manager on site, every practice can have a list of community and electronic resources related to ADHD management. A printed list of resources can be easily shared with families, and clinicians can circle relevant resources or print selected electronic resources. Phone numbers and URLs for developmental services, the school system's point of entry for special education evaluations, organizations that support families with educational advocacy, and preferred local mental health providers for children and adults should be included. One resource that families or providers can use to identify support for basic needs including housing, food, transportation, and legal resources is called Aunt Bertha, available at auntbertha.com.^66^

**Figure 7.**
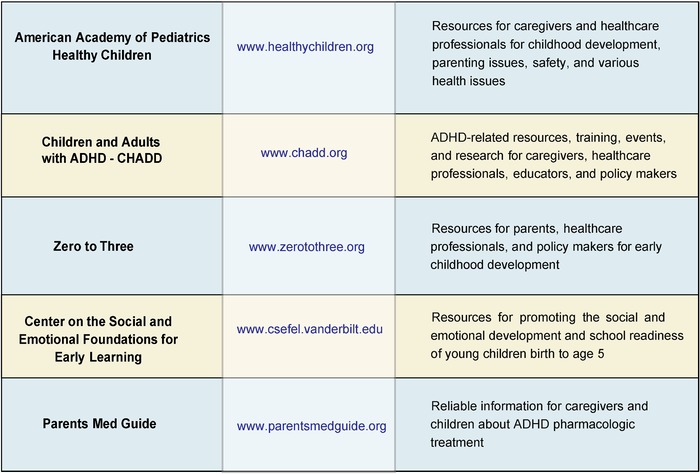
**Point of care attention-deficit/hyperactivity disorder (ADHD) and child development educational resources for caregivers and healthcare professionals.** CHADD, Children and Adults with Attention-Deficit/Hyperactivity Disorder.

Point of care educational resources for families are also valuable to reinforce the verbal guidance provided during a visit (Figure 7).

## CONCLUSION

Despite the availability of evidence-based recommendations and resources, significant opportunities exist to provide enhanced ADHD care at the primary care level, especially in Louisiana where the high prevalence of some risk factors for ADHD and the high rates of ADHD and medication prescriptions have been noted nationally and at the state level. Specialty workforce shortages, factors in the educational system, and factors associated with race and geography seem to play important roles. Attention to these factors can potentially help address these disproportionalities effectively. When the youngest children in classrooms are predictably being diagnosed with ADHD more than their older peers, questioning whether the existing classroom structure can address the needs of the entire developmental spectrum is reasonable. Mental health consultation in classrooms may be useful in supporting teachers to adapt to the wide developmental range in early elementary school. Additionally, examining if high-stakes testing and teacher evaluations based on student performance influence these patterns may be worth considering.

Specialty workforce shortages in child and adolescent psychiatry are unlikely to be fully remedied in a short time span, so innovative models of training and collaboration in pediatrics are imperative. Specifically, required training in residency, ongoing case-based learning after residency, and consultation and collaborative care are promising approaches to improving the quality of care. At the practice level, the AAP ADHD guidelines, the AAP ADHD toolkit, and scheduling recommendations offer practical approaches that can enhance the care children receive. Statewide initiatives to support innovative healthcare delivery approaches, such as consultation, could bring improved care to Louisiana's most underserved children with ADHD. Pediatric clinicians, mental health providers, and families can work together to increase awareness about the needs of children and families affected by ADHD in medical, educational, and policy arenas and move the system forward for children.

## Notes

This article meets the Accreditation Council for Graduate Medical Education and the American Board of Medical Specialties Maintenance of Certification competencies for Patient Care and Medical Knowledge.

## References

[1] American Psychiatric Association. Diagnostic and Statistical Manual of Mental Disorders: DSM-5. 5th ed Arlington, VA: American Psychiatric Publishing; 2013.

[2] Visser SN, Danielson ML, Bitsko RH, Trends in the parent-report of health care provider-diagnosed and medicated attention-deficit/hyperactivity disorder: United States, 2003-2011. J Am Acad Child Adolesc Psychiatry. 2014 1;53(1):34-46.e2. doi: 10.1016/j.jaac.2013.09.001. 24342384 PMC4473855

[3] Danielson ML, Bitsko RH, Ghandour RM, Holbrook JR, Kogan MD, Blumberg SJ Prevalence of parent-reported ADHD diagnosis and associated treatment among U.S. children and adolescents, 2016 J Clin Child Adolesc Psychol. 2018 Mar-Apr;47(2):199-212. doi: 10.1080/15374416.2017.1417860. PMC5834391 29363986

[4] Sayal K, Prasad V, Daley D, Ford T, Coghill D ADHD in children and young people: prevalence, care pathways, and service provision. Lancet Psychiatry. 2018 2;5(2):175-186. doi: 10.1016/S2215-0366(17)30167-0. 29033005

[5] Polanczyk GV, Salum GA, Sugaya LS, Caye A, Rohde LA Annual research review: a meta-analysis of the worldwide prevalence of mental disorders in children and adolescents. J Child Psychol Psychiatry. 2015 3;56(3):345-365. doi: 10.1111/jcpp.12381. 25649325

[6] Wolraich ML, McKeown RE, Visser SN, The prevalence of ADHD: its diagnosis and treatment in four school districts across two states. J Atten Disord. 2014 10;18(7):563-575. doi: 10.1177/1087054712453169. 22956714

[7] Louisiana ADHD Task Force. Prepared in response to senate concurrent resolution no. 39 of the 2014 regular session. Louisiana Department of Health and Hospitals. www.dhh.la.gov/assets/ADHD/ADHD_DHH_RspnseRsltn39.pdf. Published February 2015. Accessed February 17, 2018.

[8] Subcommittee on Attention-Deficit/Hyperactivity Disorder; Steering Committee on Quality Improvement and Management; Wolraich M, Brown L, Brown RT, ADHD: clinical practice guideline for the diagnosis, evaluation, and treatment of attention-deficit/hyperactivity disorder in children and adolescents. Pediatrics. 2011 11;128(5):1007-1022. doi: 10.1542/peds.2011-2654. 22003063 PMC4500647

[9] Barbaresi WJ. Improving care for children with ADHD: the information is just a rating scale away. Pediatrics. 2016 3;137(3):e20154450. doi: 10.1542/peds.2015-4450. 26928970

[10] Gleason MM, Egger HL, Emslie GJ, Psychopharmacological treatment for very young children: contexts and guidelines. J Am Acad Child Adolesc Psychiatry. 2007 12;46(12):1532-1572. doi: 10.1097/chi.0b013e3181570d9e. 18030077

[11] Epstein JN, Kelleher KJ, Baum R, Variability in ADHD care in community-based pediatrics. Pediatrics. 2014 12;134(6):1136-1143. doi: 10.1542/peds.2014-1500. 25367532 PMC4243070

[12] Follow-Up Care for Children Prescribed ADHD Medication (ADD). HEDIS Measures. National Committee for Quality Assurance. www.ncqa.org/hedis/measures/follow-up-care-for-children-prescribed-adhd-medication/. Accessed September 20, 2019.

[13] Fiks AG, Ross ME, Mayne SL, Preschool ADHD diagnosis and stimulant use before and after the 2011 AAP practice guideline. Pediatrics. 2016 12;138(6). pii: e20162025. doi: 10.1542/peds.2016-2025. PMC5127073 27940706

[14] State-Based Prevalence Data of Parent Reported ADHD Diagnosis by a Health Care Provider. Centers for Disease Control and Prevention. www.cdc.gov/ncbddd/adhd/prevalence.html. Accessed October 6, 2018.

[15] Sayal K, Chudal R, Hinkka-Yli-Salomäki S, Joelsson P, Sourander A Relative age within the school year and diagnosis of attention-deficit hyperactivity disorder: a nationwide population-based study. Lancet Psychiatry. 2017 11;4(11):868-875. doi: 10.1016/S2215-0366(17)30394-2. 29033006

[16] Pottegård A, Hallas J, Hernández-Díaz, Zoëga H Children's relative age in class and use of medication for ADHD: a Danish nationwide study. J Child Psychol Psychiatry. 2014 11;55(11):1244-1250. doi: 10.1111/jcpp.12243. 24813478 PMC4277337

[17] Percentage of Babies Born Low Birthweight by State. National Vital Statistics Reports. Centers for Disease Control and Prevention. www.cdc.gov/nchs/pressroom/sosmap/lbw_births/lbw.htm. Published 2018. Accessed October 10, 2019.

[18] States ranked by risk of damage from natural hazards: CoreLogic. Insurance J. www.insurancejournal.com/news/national/2014/09/10/340082.htm. Published September 10, 2014 Accessed June 19, 2018.

[19] Murder map: deadliest U.S. cities. CBS News. www.cbsnews.com/pictures/murder-map-deadliest-u-s-cities/. Published 2018. Accessed June 19, 2018.

[20] Sacks V, Murphey D. The prevalence of adverse childhood experiences, nationally, by state, and by race/ethnicity. Research Brief. files.constantcontact.com/d43ea8d3101/07cae41f-ef58-439c-b4e4-89402a31853f.pdf. Published February 2018. Accessed June 19, 2018.

[21] Louisiana Healthy Homes and Childhood Lead Poisoning Prevention Program Surveillance System Report, 2016. Louisiana Department of Health. ldh.la.gov/assets/oph/Center-PHCH/Center-PH/genetic/LEAD/SurvellianceData/2016LeadReportfinaledits04042018.pdf. Accessed October 11, 2019.

[22] Louisiana Chapter of the American Academy of Pediatrics. www.laaap.org. Accessed November 18, 2018.

[23] Workforce Maps by State. American Academy of Child and Adolescent Psychiatry. www.aacap.org/aacap/advocacy/federal_and_state_initiatives/workforce_maps/home.aspx. Accessed November 18, 2018.

[24] American Academy of Pediatrics Member Directory. American Academy of Pediatrics. www.aap.org/en-us/my-aap/directories-rosters/Pages/memberdirectory.aspx. Accessed November 18, 2018.

[25] Visser S, Holbrock J, Danielson M, Child Development Studies Team. Epidemiology of attention-deficit/hyperactivity disorder: national and state-based patterns and opportunities for policy evaluation. ldh.la.gov/assets/ADHD/ADHDSymposiumDrVisser20141209.pdf. Published December 9, 2014 Accessed September 20, 2019.

[26] Zuckerbrot RA, Cheung A, Jensen PS, Stein REK, Laraque D; GLAD-PC Steering Group. Guidelines for adolescent depression in primary care (GLAD-PC): Part I. Practice preparation, identification, assessment, and initial management. Pediatrics. 2018 3;141(3). pii: e20174081. doi: 10.1542/peds.2017-4081. 29483200

[27] Horwitz SM, Caspary G, Storfer-Isser A, Is developmental and behavioral pediatrics training related to perceived responsibility for treating mental health problems? Acad Pediatr. 2010 Jul-Aug;10(4):252-259. doi: 10.1016/j.acap.2010.03.003. 20554260

[28] Horwitz SM, Storfer-Isser A, Kerker BD, Barriers to the identification and management of psychosocial problems: changes from 2004 to 2013. Acad Pediatr. 2015 Nov-Dec;15(6):613-620. doi: 10.1016/j.acap.2015.08.006. 26409303 PMC4639452

[29] Content Outline: Developmental-Behavioral Pediatrics. American Board of Pediatrics. www.abp.org/sites/abp/files/pdf/developmental_behavioral_content_outline.pdf. Accessed June 29, 2018.

[30] McMillan JA, Land M, Leslie LK Pediatric residency education and the behavioral and mental health crisis: a call to action. Pediatrics. 2017 1;139(1). pii: e20162141. doi: 10.1542/peds.2016-2141. 28011943

[31] Frazer C, Emans SJ, Goodman E, Luoni M, Bravender T, Knight J Teaching residents about development and behavior: meeting the new challenge. Arch Pediatr Adolesc Med. 1999 11;153(11):1190-1194. doi: 10.1001/archpedi.153.11.1190. 10555724

[32] Triple Board Residency Training. American Academy of Child and Adolescent Psychiatry. www.aacap.org/AACAP/Medical_Students_and_Residents/Triple_Board_Residency_Training/Triple_Board_Residency_Training.aspx. Accessed May 10, 2019.

[33] Post Pediatric Portal Programs. American Academy of Child and Adolescent Psychiatry. www.aacap.org/AACAP/Medical_Students_and_Residents/Triple_Board_Residency_Training/Post_Pediatric_Portal_Programs.aspx. Accessed May 10, 2019.

[34] Louisiana State Loan Repayment Program. Bureau of Primary Care and Rural Health, Louisiana Department of Health. ldh.la.gov/index.cfm/page/1195. Accessed November 18, 2018.

[35] Raval GR, Doupnik SK Closing the gap: improving access to mental health care through enhanced training in residency. Pediatrics. 2017 1;139(1). pii: e20163181. doi: 10.1542/peds.2016-3181. Epub 2016 Dec 6. PMC5192092 27940515

[36] Behavioral and Mental Health. American Board of Pediatrics. www.abp.org/foundation/behavioral-mental-health. Accessed November 18, 2018.

[37] The REACH Institute. www.thereachinstitute.org. Accessed June 29, 2018.

[38] Project ECHO. University of New Mexico School of Medicine. echo.unm.edu. Accessed November 18, 2018.

[39] National Network of Child Psychiatry Access Programs. nncpap.org. Accessed November 18, 2018.

[40] Unützer J, Harbin H, Schoenbaum M, Druss B The collaborative care model: an approach for integrating physical and mental health care in Medicaid health homes. Center for Health Care Strategies, Inc. www.chcs.org/media/HH_IRC_Collaborative_Care_Model__052113_2.pdf. Published May 2013. Accessed June 20, 2018.

[41] Wright DR, Haaland WL, Ludman E, McCauley E, Lindenbaum J, Richardson LP The costs and cost-effectiveness of collaborative care for adolescents with depression in primary care settings: a randomized clinical trial. JAMA Pediatr. 2016 Nov 1;170(11):1048-1054. doi: 10.1001/jamapediatrics.2016.1721. 27654449

[42] Tulane Early Childhood Collaborative – TECC. medicine.tulane.edu/centers-institutes/tecc. Accessed November 18, 2018.

[43] Project LAUNCH Louisiana. louisianalaunch.org. Accessed November 18, 2018.

[44] Osofsky HJ, Osofsky JD, Wells JH, Weems C Integrated care: meeting mental health needs after the Gulf oil spill. Psychiatr Serv. 2014 Mar 1;65(3):280-283. doi: 10.1176/appi.ps.201300470. 24584523

[45] Myers K, Stoep AV, Thompson K, Zhou C, Unützer J Collaborative care for the treatment of Hispanic children diagnosed with attention-deficit hyperactivity disorder. Gen Hosp Psychiatry. 2010 Nov-Dec;32(6):612-614. doi: 10.1016/j.genhosppsych.2010.08.004. 21112453

[46] Kolko DJ, Campo J, Kilbourne AM, Hart J, Sakolsky D, Wisniewski S Collaborative care outcomes for pediatric behavioral health problems: a cluster randomized trial. Pediatrics. 2014 4;133(4):e981-e992. doi: 10.1542/peds.2013-2516. 24664093 PMC3966503

[47] Silverstein M, Hironaka LK, Walter HJ, Collaborative care for children with ADHD symptoms: a randomized comparative effectiveness trial. Pediatrics. 2015 4;135(4):e858-e867. doi: 10.1542/peds.2014-3221. 25802346

[48] Cheng TL, Perrin EC, DeWitt TG, O’Connor KG Use of checklists in pediatric practice. Arch Pediatr Adolesc Med. 1996 7;150(7):768-769. 8673209

[49] Gleason MM, Zeanah CH, Dickstein S Recognizing young children in need of mental health assessment: development and preliminary validity of the early childhood screening assessment. Infant Ment Health J. 2010 5;31(3):335-357. doi: 10.1002/imhj.20259. 28543224

[50] Fallucco EM, Robertson Blackmore E, Bejarano CM, Wysocki T, Kozikowski CB, Gleason MM Feasibility of screening for preschool behavioral and emotional problems in primary care using the early childhood screening assessment. Clin Pediatr (Phila). 2017 1;56(1):37-45. doi: 10.1177/0009922816638077. 27009614

[51] Larson K, Russ SA, Kahn RS, Halfon N Patterns of comorbidity, functioning, and service use for US children with ADHD, 2007 Pediatrics. 2011 3;127(3):462-470. doi: 10.1542/peds.2010-0165. PMC3065146 21300675

[52] Wolraich ML, Bard DE, Neas B, Doffing M, Beck L The psychometric properties of the Vanderbilt attention-deficit hyperactivity disorder diagnostic teacher rating scale in a community population. J Dev Behav Pediatr. 2013 2;34(2):83-93. doi: 10.1097/DBP.0b013e31827d55c3. 23363973

[53] Pediatric Symptom Checklist-17. www.prohealthmd.com/windhampediatrics-dev/wp-content/uploads/sites/25/2015/01/PSC-17.pdf. Accessed October 6, 2019.

[54] Gardner W, Lucas A, Kolko DJ, Campo JV Comparison of the PSC-17 and alternative mental health screens in an at-risk primary care sample. J Am Acad Child Adolesc Psychiatry. 2007 5;46(5):611-618. doi: 10.1097/chi.0b013e318032384b. 17450052

[55] Johnson JG, Harris ES, Spitzer RL, Williams JB The patient health questionnaire for adolescents: validation of an instrument for the assessment of mental disorders among adolescent primary care patients. J Adolesc Health. 2002 3;30(3):196-204. 11869927 10.1016/s1054-139x(01)00333-0

[56] Lewandowski RE, O’Connor B, Bertagnolli A, Screening for and diagnosis of depression among adolescents in a large health maintenance organization. Psychiatr Serv. 2016 Jun 1;67(6):636-641. doi: 10.1176/appi.ps.201400465. 26876655 PMC5556930

[57] Birmaher B, Brent DA, Chiappetta L, Bridge J, Monga S, Baugher M Psychometric properties of the Screen for Child Anxiety Related Emotional Disorders (SCARED): a replication study. J Am Acad Child Adolesc Psychiatry. 1999 10;38(10):1230-1236. doi: 10.1097/00004583-199910000-00011. 10517055

[58] Instruments: Screen for Child Anxiety Related Emotional Disorders (SCARED). University of Pittsburgh. www.pediatricbipolar.pitt.edu/resources/instruments. Accessed October 6, 2019.

[59] Dubowitz H, Lane WG, Semiatin JN, Magder LS, Venepally M, Jans M The safe environment for every kid model: impact on pediatric primary care professionals. Pediatrics. 2011 4;127(4):e962-e970. doi: 10.1542/PEDS.2010-1845. 21444590 PMC3387892

[60] Garg A, Butz AM, Dworkin PH, Lewis RA, Thompson RE, Serwint JR Improving the management of family psychosocial problems at low-income children's well-child care visits: the WE CARE project. Pediatrics. 2007 9;120(3):547-558. doi: 10.1542/peds.2007-0398. 17766528

[61] Purewal SK, Bucci M, Gutiérrez L, Screening for adverse childhood experiences (ACEs) in an integrated pediatric care model. pediatriesociale.fondationdrjulien.org/wp-content/uploads/2017/05/2016-01-Purewal_ace.pdf. Published January 2016. Accessed November 18, 2018.

[62] SEEK Plus – Tulane Early Childhood Collaborative. medicine.tulane.edu/sites/medicine.tulane.edu/files/Seek plus.pdf. Accessed November 18, 2018.

[63] Screening Technical Assistance & Resource (STAR) Center. American Academy of Pediatrics. www.aap.org/en-us/advocacy-and-policy/aap-health-initiatives/Screening/Pages/default.aspx. Accessed October 6, 2019.

[64] Screening Tools Finder. STAR Center. American Academy of Pediatrics. screeningtime.org/star-center/#/screening-tools#top. Accessed October 6, 2019.

[65] A 14-month randomized clinical trial of treatment strategies for attention-deficit/hyperactivity disorder. The MTA Cooperative Group. Multimodal Treatment Study of Children with ADHD. Arch Gen Psychiatry. 1999 12;56(12):1073-1086. 10591283 10.1001/archpsyc.56.12.1073

[66] Aunt Bertha – Connecting People and Programs. www.auntbertha.com. Accessed November 18, 2018.

